# Renal histology in diabetic nephropathy predicts progression to end-stage kidney disease but not the rate of renal function decline

**DOI:** 10.1186/s12882-020-01943-1

**Published:** 2020-07-18

**Authors:** Paraish S. Misra, Stephen G. Szeto, Adriana Krizova, Richard E. Gilbert, Darren A. Yuen

**Affiliations:** 1 Division of Nephrology, St. Michael’s Hospital, Unity Health Toronto and University of Toronto, 30 Bond St, Toronto, ON M5B 1W8 Canada; 2 Department of Pathology and Laboratory Medicine, St. Michael’s Hospital, Unity Health Toronto and University of Toronto, 30 Bond St, Toronto, ON M5B 1W8 Canada; 3 Division of Endocrinology, St. Michael’s Hospital, Unity Health Toronto and University of Toronto, 30 Bond St, Toronto, ON M5B 1W8 Canada; 4 Keenan Research Centre for Biomedical Science, Li Ka Shing Knowledge Institute, St. Michael’s Hospital, Rm 509, 5th Floor, 209 Victoria Street, Toronto, ON M5B 1T8 Canada

**Keywords:** Diabetic nephropathy, eGFR slope, Renal Pathology Society, Kidney biopsy, ESKD

## Abstract

**Background:**

While histopathologic changes correlate with functional impairment in cross-sectional studies of diabetic nephropathy (DN), whether these findings predict future rate of kidney function loss remains uncertain. We thus sought to examine the relationship between kidney histopathology, incidence of end-stage kidney disease (ESKD), and rate of estimated glomerular filtration rate (eGFR) loss in DN.

**Methods:**

In this longitudinal cohort study, we studied 50 adults diagnosed with biopsy-proven DN. We analyzed the histopathologic parameters of each patient’s kidney biopsy, as defined by the Renal Pathology Society classification system for DN, and tracked all available eGFR measurements post-biopsy. We additionally collected baseline clinical parameters (at the time of biopsy), including eGFR, albumin-to-creatinine ratio (ACR), and hemoglobin A_1c_. Multivariable linear regression was used to assess the relationship between histologic and clinical parameters at the time of the biopsy and eGFR slope. Kaplan-Meier curves and Cox regression were used to evaluate the association between histologic and clinical parameters and ESKD incidence.

**Results:**

Progression to ESKD was associated with worsening interstitial fibrosis score (*p* = 0**.**05), lower baseline eGFR (*p* = 0**.**02), higher ACR (*p* = 0**.**001), and faster eGFR decline (*p* < 0.001). The rate of eGFR decline did not associate with any histologic parameter. Baseline ACR was the only studied variable correlating with eGFR slope (rho = − 0**.**41).

**Conclusions:**

Renal histology predicts ultimate progression to ESKD, but not the rate of progression. Future work is required to identify novel predictors of rapid functional decline in patients with diabetic nephropathy.

## Background

With nearly one in every ten Americans diagnosed with diabetes, diabetic nephropathy (DN) has become the most common cause of kidney failure in the United States [1, 2]. Despite this alarming statistic, most patients with diabetes do not develop kidney failure, and in fact many develop only mild renal dysfunction [3, 4].

eGFR is a key marker of kidney function, and in steady state reflects the degree of established chronic kidney disease. The rate of eGFR change, on the other hand, is a powerful clinical measure of DN progression. Recent evidence suggests that eGFR declines linearly in the majority of patients with diabetic nephropathy, although the rate may vary significantly between patients [5–7]. While a number of high risk clinical features such as hypertension, high-grade albuminuria, and poor glycemic control are well established [6, 8], our ability to measure the rate of kidney function loss in DN and identify “rapid progressors” remains limited [9]. Understanding the factors that influence rate of eGFR decline could help predict the clinical course for individual patients and also help identify rapid progressors for studies of novel renoprotective agents.

Renal biopsy, although rarely performed for the evaluation of pure DN, could provide important structural information that might correlate with the rate of eGFR loss. In 2010, the Renal Pathological Society (RPS) devised a classification system for DN to standardize inter-biopsy comparisons, with separate categories for glomerular, interstitial, and vascular lesions [10]. Early validation studies suggested that higher-grade lesions, as assessed by the RPS classification, are associated with a greater risk of end-stage kidney disease (ESKD) and need for dialysis [11–17]. However, whether renal histology simply reflects established disease, which can be assessed non-invasively, or also can predict future rate of eGFR decline, remains unknown. Our aim was to determine whether the 2010 RPS histologic classification system outperformed clinical parameters in predicting the rate of post-biopsy eGFR loss and progression to ESKD in diabetic nephropathy.

## Methods

### Patients and study design

The St. Michael’s Hospital Research Ethics Board approved the study protocol (REB 16–118), which adhered to the Declaration of Helsinki. Patients were included if they had a diagnosis of type 1 or type 2 diabetes, and had a native kidney biopsy at St. Michael’s Hospital between 2010 and 2017 that demonstrated either pure DN or features of non-specific vascular disease, including glomerulosclerosis, non-inflammatory vascular disease, or interstitial fibrosis and tubular atrophy. To ensure consistency of histopathologic assessment, biopsies were re-evaluated according to the 2010 RPS classification by a single renal pathologist (A.K.) blinded to clinical data, including rate of change of eGFR over time. Each biopsy was evaluated by light microscopy, immunofluorescence (using sections stained for IgG, IgM, IgA, C3, C1q, kappa and lambda) and electron microscopy. Baseline clinical information and longitudinal laboratory data were then collected for each patient and stored in a de-identified summary format. Patients were excluded if they had less than 6 months of follow-up and less than three follow-up data points, if they had no clinical documentation or biochemical evidence of diabetes mellitus, or if their biopsies demonstrated non-diabetic pathology on re-evaluation by the renal pathologist.

### Baseline and follow-up clinical data and definitions

For each patient, the diagnosis of diabetes mellitus (either type 1 or 2) was ascertained from the treating nephrologist’s or endocrinologist’s clinical notes, or the presence of two serum HbA_1c_ results greater than 6**.**5% tested on different days, consistent with the 2017 American Diabetes Association guidelines [18]. The following baseline clinical data were collected: age, sex, eGFR as calculated by the CKD Epidemiology Collaboration formula (with serum creatinine, sex, and age) [19], urinary ACR, HbA_1c_, number of oral hypoglycemic medications, insulin therapy, hemoglobin level at biopsy, systolic (SBP) and diastolic blood pressures (DBP), number of anti-hypertensive medications, renin-angiotensin system (RAS) blockade therapy, LDL levels, HDL levels, and lipid lowering therapy. Post-biopsy follow-up data was collected for eGFR, ACR, and HbA_1c_. All laboratory tests were performed by the clinical biochemistry laboratory at St. Michael’s Hospital. Baseline was defined as the date at which data were available closest to and prior to the biopsy, and follow-up data were collected starting from the date of biopsy until the patient was either lost to follow-up or met the study end-point (ESKD).

### Histologic review using Renal Pathology Society classification system

Each biopsy was reviewed by a single pathologist (A.K.) according to the RPS classification system [10]. Using this system, each biopsy was assessed for 3 types of lesions: glomerular, interstitial, and vascular. Glomerular lesions were classified into one of four classes: (a) mild or nonspecific light microscopic changes and electron-microscopy proven glomerular basement membrane thickening (class I), (b) mesangial expansion (class II), (c) nodular sclerosis (class III), and (d) advanced diabetic glomerulosclerosis (class IV). Similarly, interstitial fibrosis and tubular atrophy (IF/TA), interstitial inflammation, arteriolar hyalinosis, and large vessel arteriosclerosis were quantified on categorical scales ranging between 0 and 3 (IF/TA) or 0–2 (other variables). Data were stored in a de-identified, summary format.

### Determination of progressor class

Slopes of eGFR change over time were calculated by applying a regression model on post-biopsy eGFR data. A priori, patients were categorized into the following eGFR progressor classes, as defined previously [5]: slow (eGFR decline of less than 5 ml/min/year), moderate (eGFR decline between 5 and 10 ml/min/year), and fast (eGFR decline greater than 10 ml/min/year). GFR slope was calculated in absolute (eGFR change in ml/min/year) rather than relative terms (eGFR change in ml/min/year divided by eGFR at time of initial follow-up, expressed as a percentage) to avoid undue slope inflation for patients with lower eGFR values at study entry. A negative value for eGFR slope indicates eGFR loss over time, and thus a greater (or more positive) slope indicates slower eGFR loss, while a numerically lower (or more negative) slope indicates faster eGFR loss.

### ESKD definition and survival curves

ESKD was defined as the need for dialysis initiation or transplantation. Renal survival was defined as the absence of ESKD during follow-up. For Kaplan-Meier survival curves, patients were divided into two groups according to histology classification (as defined in the text, Tables, and Figure legends; see Additional file 1, Supplementary Table 1 for more details), eGFR progressor class, eGFR at biopsy, ACR at biopsy, and HbA_1c_ at biopsy. For clinical parameters, patients were divided into two groups by the median value to optimize power for statistical analyses. Decline to an eGFR below 15 ml/min was included in the outcome of ESKD as a sensitivity analysis.

### Statistical analysis

Mann-Whitney U and Kruskal-Wallis tests were used to calculate differences in variable distributions for comparisons of two and greater than two covariates, respectively. Spearman’s correlation analysis was used to assess the relationships between baseline clinical parameters, histologic parameters, and slope of eGFR change. Kaplan–Meier survival curves were generated for histologic and clinical parameters, and the logrank (Mantel-Cox) test was used to test for differences between groups and adjust for confounding variables. Glomerular classes IIA and IIB were combined for all analyses. The RPS glomerular classification and all RPS histologic scores were treated as scales of increasing severity. For survival and regression analyses, histology scores were reclassified into binary categorical variables (“low” vs “high”) to optimize the balance between group size and number of events per group (see Additional file 1, Supplementary Table 1). No method of data imputation was used for missing data. All analyses were performed in GraphPad Prism (version 6**.**01, Graphpad Software Inc.) or R (version 3**.**4**.**2, The R Foundation for Statistical Computing).

## Results

### Patient enrollment

**Fig. 1:**
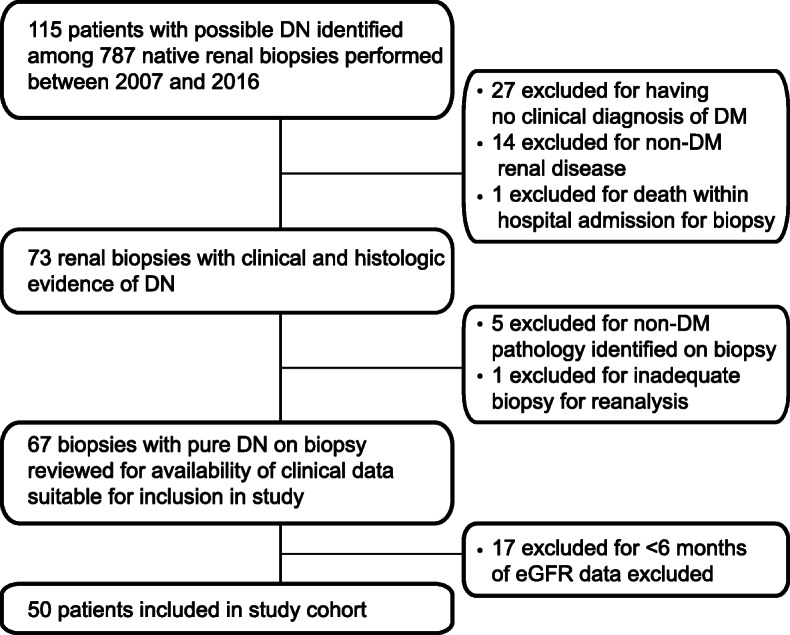
CONSORT diagram for patient inclusion and exclusion. Among 787 native kidney biopsies performed at St. Michael’s Hospital, a quaternary care hospital in Toronto, Canada, 50 unique cases of pure diabetic nephropathy with appropriate follow–up clinical data were identified for inclusion in our study. DN, diabetic nephropathy; eGFR, estimated glomerular filtration rate. DN, diabetic nephropathy; DM, diabetes mellitus. [BLACK AND WHITE]

Among 787 patients that underwent native kidney biopsy between January 2007 and September 2016 at St. Michael’s Hospital, 73 had a clinical diagnosis of diabetes mellitus and a pathologic diagnosis of DN. Of these, 50 patients were enrolled in the study. Details of the patient selection process are outlined in Fig. 1. The most common indications for renal biopsy among patients included in the study were assessment of proteinuria (40%), followed by elevated or subacute rise in serum creatinine levels (18%, see Additional file 1, Supplementary Table 2).

### Cohort characteristics

Data for the entire cohort is summarized in Table 1. The median age of study participants was 56 years, and 64% were male. The median eGFR and urinary ACR ratio at time of biopsy were 43 mL/min and 216 mg/mmol, respectively. Over half of all biopsies had either Kimmelstiel-Wilson lesions or advanced diabetic glomerulosclerosis, as well as moderate or severe interstitial fibrosis/tubular atrophy (IF/TA). Similarly, over half of all biopsies demonstrated more than one area of arteriolar hyalinosis.

**Table 1:** Baseline clinical and histologic information for entire cohort, stratified by progressor class

Progressor class	All	Slow progressors	Moderate progressors	Fast progressors	***P*** value
No. of patients (%)	50 (100)	20 (40)	16 (32)	14 (28)	
eGFR slope, ml/min/yr (med, IQR)	−5.8 (−12.4, −3.1)	−2.3 (− 3.6,1.0)	−6.5 (−7.8–5.7)	−17.4 (− 28.2, − 13.9)	**< 0.001**
FU data points (med, IQR)	13 (8, − 17.75)	13 (12,18)	14 (11,21)	9.5 (7.2,14.2)	0.2
FU time,years (med, IQR)	2.53 (1.22,3.65)	2.81 (2.39,4.49)	2.8 (2.03,4.35)	1.13 (0.88, 2.43)	**0.01**
Sex male (n,%)	32 (64)	14 (70)	10 (62.5)	8 (57.1)	0.7
Age,years (med, IQR)	56 (47.25–62.75)	58 (54.8,64.2)	52.5 (46.8 59.2)	50 (46.2,58.5)	0.2
eGFR,mls/min (med, IQR)	43.1 (28.7,52.6)	37.2 (19.7,51.2)	38.7 (28.1–67.7)	45.3 (41.4, 52)	0.3
ACR, mg/mmol (med, IQR)	216 (114,477)	173 (70.6212.5)	260 (121,581)	742 (284,999)	**0.004**
HbA_1c_, % (med, IQR)	7.4 (6.6–9.1)	6.8 (6.4,7.5)	7.6 (6.8,8.9)	9.0 (6.8,11.8)	**0.05**
Use of insulin (n, %)	33 (66)	11 (55)	13 (81.2)	9 (64.3)	0.3
#OHG (mean)	0.9	1.15	0.75	0.714	0.4
SBP, mmHg (med, IQR)	156 (141–172)	151 (141,172)	154 (132–171)	157 (146,165)	0.9
#Anti-HTN (mean)	3.32	3.1	3.44	3.5	0.8
Use of RASi (n,%)	39 (78)	16 (80)	13 (81.2)	10 (71.4)	0.8
Use of statin (n,%)	39 (78)	15 (75)	14 (87.5)	10 (71.4)	0.5
Hb (mg/L, IQR)	110 (96.5122.2)	109 (96.5–120)	111 (99.75–127)	110 (98.25–117.25)	0.9
Renal death (n,%)	20 (40)	4 (20)	7 (43.8)	9 (64.3)	**0.03**
Glomerular Class (%)					0.9
I	2 (4.0)	1 (5.3)	1 (6.3)	0 (0)	
II	7 (14.3)	3 (15.8)	2 (12.5)	2 (14.2)	
III	33 (67.3)	12 (63.2)	11 (68.8)	10 (71.4)	
IV	7 (14.3)	3 (15.8)	2 (12.5)	2 (14.3)	
IF/TA Score %					0.9
0	0 (0)	0 (0)	0 (0)	0 (0)	
1	16 (32)	6 (30)	5 (31.3)	5 (35.7)	
2	20 (40)	8 (40)	8 (50)	4 (28.6)	
3	14 (28)	6 (30)	3 (18.8)	5 (35.7)	
Arteriolar Hyalinosis Score %					0.6
0	4 (8)	2 (10)	2 (12.5)	0 (0)	
1	14 (28)	5 (25)	5 (31.3)	4 (28.6)	
2	32 (64)	13 (65)	9 (56.3)	10 (71.4)	
Arteriosclerosis Score %					0.4
0	2 (4.2)	1 (5.3)	0 (0)	1 (7.1)	
1	21 (44.7)	10 (52.6)	5 (35.7)	6 (42.9)	
2	24 (51.1)	8 (42.1)	9 (64.3)	7 (50)	

Slow, medium and fast progressors were defined by slope of eGFR decline, as described in the Methods section: slow (eGFR decline of less than 5 ml/min/year), moderate (eGFR decline between 5 to 10 ml/min/year), and fast (eGFR decline greater 10 ml/min/year). Legend: *FU* Follow-up, *eGFR* Estimated glomerular filtration rate, *ACR* Urinary albumin to creatinine ratio, *HbA*_*1c*_ Hemoglobin A_1c_, *OHG* Oral hypoglycemic medications, *SBP* Systolic blood pressure, *HTN* Hypertension, *RASi* Renin-angiotensin system inhibitors, *Hb* Hemoglobin, *IF/TA* Interstitial fibrosis and tubular atrophy, *Med* Median, *IQR* Interquartile range

**Fig. 2:**
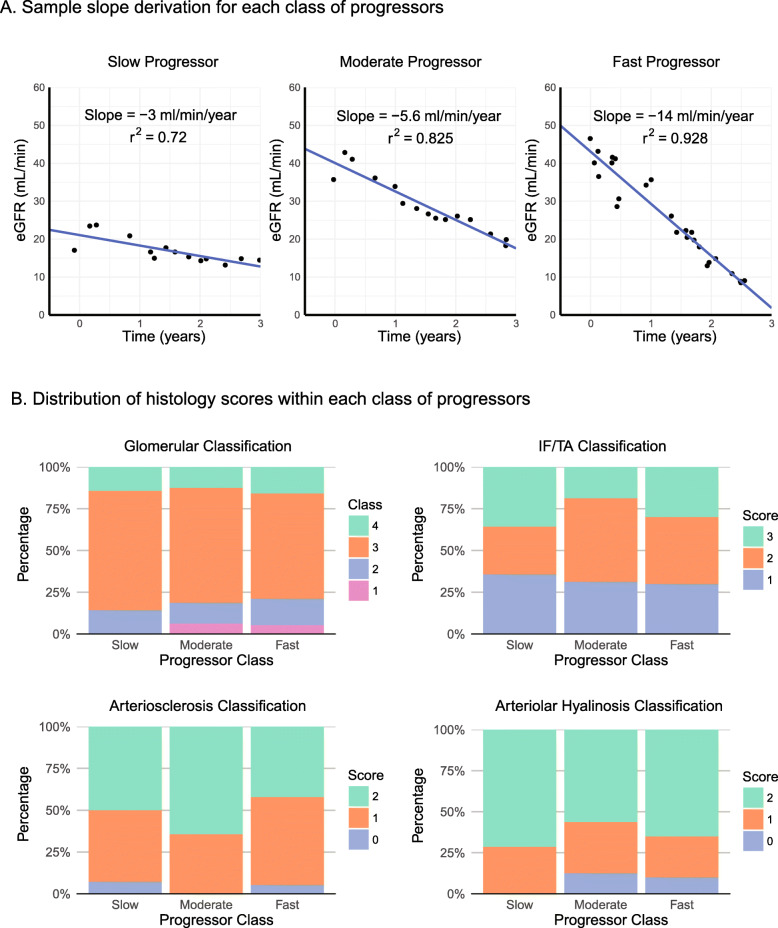
Classification into progressor classes by eGFR slope. **a**, Examples of linear regression modeling of eGFR vs. time for determination of eGFR slope. One example is given for each of the slow (eGFR slope > − 5 ml/min/year), moderate (slope − 10 to − 5 ml/min/year), and fast (slope ≤ − 10 ml/min/year) progressor classes. **b**, Stacked bar chart of distribution of histologic scores among different progressor classes. The distribution of histology scores for each category of the 2010 RPS classification system was similar between progressor classes (*p* values for glomerular, IF/TA, arteriosclerosis, and arteriolar hyalinosis scores of 0**.**93, 0**.**88, 0**.**59, and 0**.**41, respectively). eGFR, estimated glomerular filtration rate; IF/TA, interstitial fibrosis and tubular atrophy; RPS, Renal Pathology Society. [IN COLOUR]

Post-biopsy slopes of eGFR decline were calculated for each of the 50 patients as illustrated in Fig. 2a. 40, 32, 28% were slow (eGFR slope decline of less than 5 ml/min/year), moderate (eGFR decline between 5 and 10 ml/min/year), and fast progressors (greater than 10 ml/min/year decline in eGFR), respectively. Progressor groups differed significantly in eGFR slope (*p* < 0**.**001), years of follow-up (*p* = 0**.**008), urinary albumin-to-creatinine ratio (ACR) at biopsy (*p* = 0**.**004), and rates of ESKD (*p* = 0**.**03). There were no significant differences in histology scores between progressor classes (see Fig. 2b). As all patients had interstitial inflammation scores of 1, this histology classification could not be included in our analyses. Glomerular class could not be assessed in one patient, and arteriosclerosis could not be assessed in three patients due to a lack of sufficient tissue for analysis.

### Higher IF/TA scores are associated with higher rates of ESKD, but do not improve risk estimations based on serial eGFR measurements

**Fig. 3:**
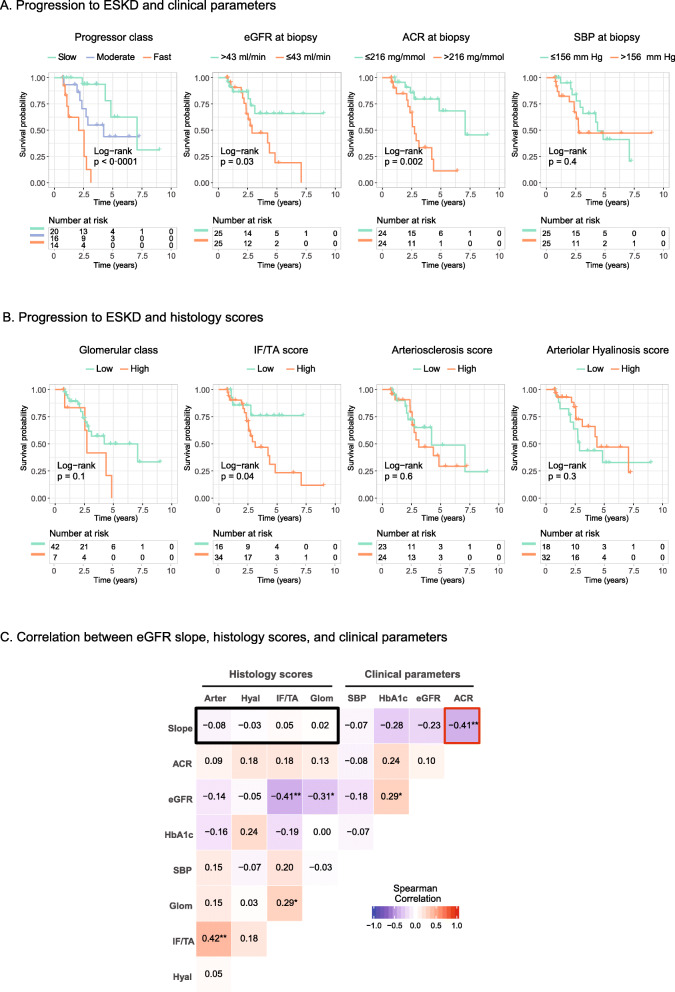
The relationship between ESKD, clinical parameters and histology scores. **a** and **b**, Kaplan-Meier curves for ESKD–free survival, stratified by clinical and histologic parameters. Statistically significant differences in survival were found for eGFR slope, eGFR at time of biopsy, ACR, and IF/TA scores. **c**, Heat map depicting the correlation between clinical and histologic parameters. Colour hue and intensity represent the direction and magnitude, respectively, of the Spearman’s rho correlation coefficient. ACR was the only parameter that correlated significantly with eGFR slope (red box). No correlation was found between histologic parameters and eGFR slope (black box). For these analyses, RPS histology categories were reclassified into binary groups as follows: Glomerular class: Low, RPS classes 1–3; High, RPS class 4. IF/TA score: Low, score 1; High, scores 2–3. Arteriosclerosis score: Low, scores 0–1; High, score 2. Arteriolar hyalinosis score: Low, scores 0–1; High, score 2. eGFR, estimated glomerular filtration rate; ACR, urinary albumin-to-creatinine ratio; SBP, systolic blood pressure; IF/TA, interstitial fibrosis and tubular atrophy; ESKD, end-stage kidney disease; Arter, arteriosclerosis score; Hyal, arteriolar hyalinosis score; Glom, glomerular class; SBP, systolic blood pressure; HbA_1c_, hemoglobin A_1c_. [IN COLOUR]

To begin characterizing the prognostic role of the RPS classification system, we first compared clinical and histologic parameters for their association with progression to ESKD. Among clinical parameters, the greatest risk of ESKD on Kaplan-Meier analysis was seen in patients with lower baseline eGFR (*p* = 0**.**03), more rapid slope of eGFR decline (*p* < 0**.**001) and higher urinary ACR at biopsy (*p* = 0**.**002), as shown in Fig. 3a. Among histologic parameters, only higher IF/TA scores associated with higher rates of ESKD (*p* = 0**.**04).

To quantify the impact of each parameter on renal survival, Cox proportional hazards regression was performed. Consistent with the above analyses, eGFR at biopsy, ACR at biopsy, HbA_1c_, IF/TA scores and eGFR slope showed significant hazard ratios for prediction of ESKD, with the greatest predictor being slope of eGFR decline (R^2^ = 0**.**26, see Table 2). The five parameters with statistical significance on univariate analysis were next included in a multivariable Cox regression model. After including slope of eGFR decline, inclusion of ACR and HbA_1c_ did not lead to significant changes in the fit of the regression model, suggesting correlation between these parameters. Similarly, inclusion of IF/TA scores did not improve any model that already included eGFR at biopsy. Thus, the model that best predicted ESKD development included only eGFR at biopsy and eGFR slope (R^2^ = 0.49, see Table 2 for final model and Additional file 1, Supplementary Table 3 for alternative models explored). Inclusion of decline to an eGFR below 15 ml/min in the outcome of ESKD as a sensitivity analysis increased the total number of events, but did not affect the results of the above analyses (see Additional file 1, Supplementary Tables 4–6). Taken together, these results confirm that both RPS histology scores and traditional clinical risk factors such as ACR and HbA_1c_ are helpful in predicting ESKD risk. However, they also suggest that ESKD risk may be best captured by a simple collection of eGFR measurements over time to enable an estimation of a patient’s absolute degree of functional impairment (i.e. established disease burden) as well as their trajectory of ongoing eGFR loss (i.e. rate of disease progression).

**Table 2:** Cox proportional hazards regression

	Covariate	HR	95% Confidence interval	***P*** value	R^**2**^
Univariate analysis	Age (per year)	1.01	0.97–1.06	0.5	0.009
	Male Sex	1.92	0.69–5.29	0.2	0.034
	Slope -Per 5 ml/min/yr decrease	1.60	1.28–2.00	**< 0.001**	0.26
	eGFR at biopsy -Per 30 ml/min decrease	2.10	1.10–4.01	**0.02**	0.11
	ACR -Per 100 mg/mmol increase	1.22	1.08–1.36	**0.001**	0.19
	Hemoglobin A_1C_ -Per 1% increase	1.12	0.96–1.31	0.1	0.04
	SBP -Per 10 mmHg increase	1.08	0.88–1.33	0.5	0.01
	Glom class (reference: Low) -High	1.67	0.60–4.69	0.3	0.02
	IF/TA (reference: Low) -High	3.43	1.00–11.8	**0.05**	0.10
	Arteriosclerosis (reference: Low) -High	1.21	0.49–2.97	0.7	0.003
	Arteriolar Hyalinosis (reference: Low) -High	0.64	0.27–1.56	0.3	0.02
Final multivariable model (*p* value < 0.001)	Slope -Per 5 ml/min/yr decrease	6.48	2.29–18.3	**< 0.001**	0.49
	eGFR at biopsy -Per 30 ml/min decrease	2.15	1.59–2.92	**< 0.001**	

Legend: *HR* Hazard ratio, *eGFR* Estimated glomerular filtration rate, *ACR* Urinary albumin to creatinine ratio, *SBP* Systolic blood pressure, *IF/TA* Interstitial fibrosis and tubular atrophy

### Traditional clinical factors are poor predictors of rate of eGFR decline, and histologic parameters offer no additive benefit

**Fig. 4:**
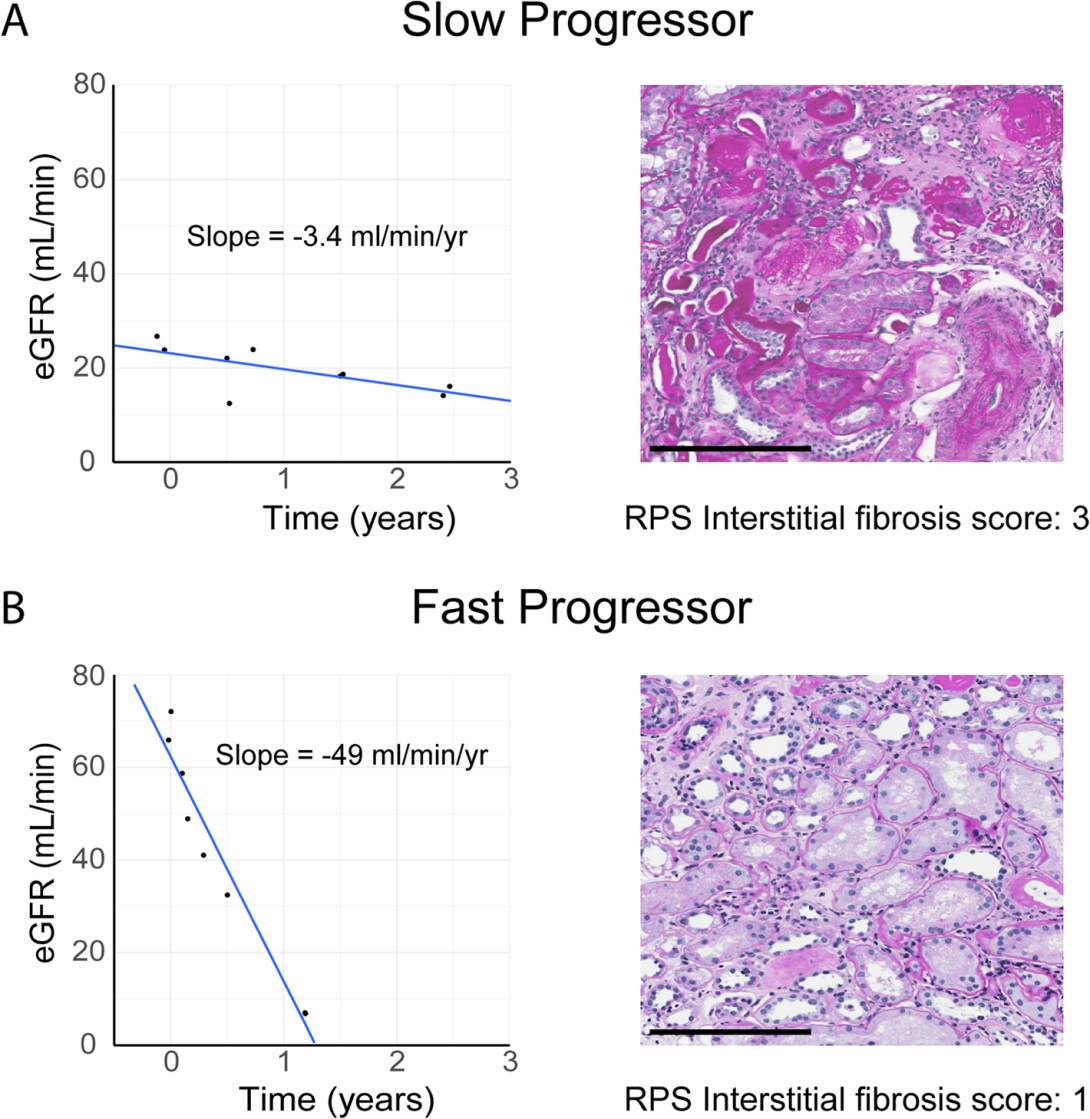
Examples of discordance between histology and eGFR slopes. Several patients in our cohort had eGFR trajectories that were highly discordant with the severity of histologic findings, highlighting the lack of correlation between eGFR slope and histology. Panel **a** contrasts the eGFR slope of a slow progressor with advanced interstitial fibrosis, while panel **b** demonstrates rapid eGFR decline despite mild interstitial fibrosis. eGFR, estimated glomerular filtration rate; RPS, Renal Pathology Society. Scale bar, 200 μm. [IN COLOUR]

While rate of eGFR decline is important in predicting ESKD risk, it requires serial eGFR measurements collected over time. Clinical and/or histologic risk factors that could help identify patients with low or high rates of eGFR loss at their first clinic visit would thus be very helpful. To address this issue, Spearman’s rank correlation coefficients were calculated between post-biopsy eGFR slope and individual clinical or histologic parameters at the time of biopsy (see Fig. 3c). As suggested by the multivariable Cox regression, eGFR slope demonstrated a significant correlation with ACR (rho = − 0**.**41, *p* = 0**.**004), and a borderline correlation with HbA_1c_ (rho = − 0**.**28, *p* = 0**.**06). Meanwhile, no significant correlations were found between eGFR slope and any histologic parameter. In fact, striking discordance was noted between histopathologic findings and eGFR slope in several patients in our cohort (see Fig. 4).

We then performed simple and multiple linear regression to quantify predictors of eGFR slope and to adjust for potential confounding. On simple regression, only ACR (*p* = 0**.**005) and HbA_1c_ (*p* = 0**.**007) were significant covariates (see Table 3). These two parameters were then included in a multiple regression model, which accounted for variance in slope to only a modest degree (R^2^ = 0**.**21, *p* = 0**.**002, see Table 3). Again, no histologic parameter predicted eGFR slope on simple regression, and thus these were not assessed for inclusion in the multivariable model.

**Table 3:** Simple linear regression for eGFR slope

	Parameter	Coefficient	Confidence interval	***P*** value	***R***^**2**^
Univariate analysis	Age -Per 10 year increase	2.51	-0.43 – 5.44	0.1	0.06
	Male Sex	1.05	-5.21 – 7.30	0.7	0.002
	eGFR at biopsy -Per 1ml/min increase	-0.09	-0.22 – 0.46	0.2	0.03
	ACR -Per 100 mg/mmol increase)	-1.24	-2.06 – 0.42	**0.005**	0.16
	HbA_1c_ -Per 1% increase	-1.60	-2.71 – -0.49	**0.007**	0.14
	SBP -Per 10 mmHg increase	-0.21	-1.64 – 1.21	0.8	0.002
	Glomerular class (reference: Low) -High	1.49	-6.70 – 9.67	0.7	0.003
	IF/TA score (reference: Low) -High	3.26	-3.12 – 9.63	0.3	0.02
	Arteriolar Hyalinosis score (reference: Low) -High	-0.62	-6.64 – 5.41	0.8	0.0008
	Arteriosclerosis score (reference: Low) -High	0.408	-5.85 – 6.67	0.9	0.0003
Final multivariable model (p = 0.002)	ACR -Per 100mg/mmol increase	-0.87	-1.73 – 0.009	**0.05**	0.21
	HbA_1C_ -Per 1% increase	-1.34	-2.51 - -0.18	**0.03**	

Legend: *eGFR* estimated glomerular filtration rate, *ACR* urinary albumin-to-creatinine ratio, *HbA*_*1c*_ hemoglobin A_1c_, *SBP* systolic blood pressure, *IF/TA* interstitial fibrosis and tubular atrophy

Our observation that IF/TA scores correlated with ESKD incidence but not eGFR slope suggested that chronic fibrotic injury seen on biopsy may simply reflect established disease, but not the rate of disease progression. Indeed, both IF/TA and glomerular injury classes correlated closely with baseline eGFR (rho = − 0**.**41 and − 0**.**31, respectively, see Fig. 3c). Taken together, these results suggest that: (1) RPS histology injury scores do not predict future rate of eGFR loss, and (2) even standard clinical parameters such as ACR and HbA_1c_ are only modestly associated with rate of eGFR loss.

## Discussion

A new paradigm in diabetic nephropathy is emerging, with progressive renal decline, and not albuminuria, as the defining feature of disease progression [5]. Although several attempts have been made to identify clinical parameters associated with the rate of eGFR loss, the role of histology in predicting DN progression remains incompletely defined. In 2010, the Renal Pathology Society devised a classification system for diabetic kidney biopsies, to help standardize the way in which such biopsies are reported. We report here that histology, as classified by this system, does not predict the post-biopsy rate of eGFR loss in diabetic nephropathy, an observation that to our knowledge has never before been described.

Previous studies have examined the relationship between the 2010 RPS classification system and DN prognosis [11–13, 15–17]. These reports focused only on the binary outcome of renal survival, finding that greater glomerular and interstitial damage was associated with higher rates of ESKD. In contrast to these studies, Mottl et al found no association between the RPS classification system and time to ESKD, attributing this discrepancy to differences in ethnic background and care practices between their population and those of prior studies [20]. Our study provides a potential resolution to this conflicting literature. We considered the outcomes of renal survival and eGFR change over time separately, and unexpectedly found that IF/TA scores were associated with progression to ESKD, but did not predict the rate of eGFR loss. This apparent paradox is likely explained by the fact that IF/TA reflects the degree of established renal injury, as suggested by its correlation with eGFR at the time of biopsy. We thus propose that IF/TA scores provide information on “disease stage”, but do not predict rate of disease progression.

The only other study to examine the association between RPS histology scores and eGFR slope was performed in a Japanese DN cohort, and reported results that were largely similar to ours [14]. Indeed, in this study, glomerular class did not predict rate of eGFR decline, nor did parameters of vascular injury or interstitial inflammation. Interestingly, only severe IF/TA (class 3), but not mild or moderate IF/TA, associated with more rapid eGFR decline in a multivariate analysis. In the bulk of their analyses, Mise et al. calculated eGFR slope as a percentage relative to initial eGFR, which itself may have been correlated to the degree of IF/TA, as seen in our study [14]. Moreover, the Japanese cohort consisted solely of patients with microalbuminuria [14], whereas our patients came from a variety of ethnic backgrounds and included those with normo-, micro-, and macroalbuminuria. As ethnicity and albuminuria are both important determinants of renal outcomes in DN [21–24], it is possible that these differences may explain the discrepancy in findings.

The only published study relating absolute eGFR slope to histology (using a non-RPS classification system) was performed on a small Japanese cohort of mostly slow progressors (37 patients) [25], finding a slightly higher eGFR decline in patients with glomerular lesions on biopsy compared to patients with minimal histologic changes. Counterintuitively, patients with the worst interstitial and vascular changes had seemingly preserved eGFR trajectories, conflicting with the results of the study by Mise et al. Interestingly, these relationships between glomerular, interstitial and vascular injury and absolute eGFR slope were not reproduced in our larger cohort of patients that included a greater representation of rapid progressors.

Our study has several important limitations. First, our relatively small sample size limits the power of our statistical analyses, and our results must thus be taken as preliminary. A larger study would have permitted the examination of important subgroups, such as type 1 vs. type 2 diabetes and hypertensive vs. non-hypertensive patients (of which there were only 8 in our study), and would have allowed a more detailed study of histologic lesions, rather than re-grouping the pathology scores into binary variables. However, as patients with DN are rarely biopsied unless another concerning feature is present (such as high grade proteinuria, rapid rate of GFR loss, or active urinary sediment), this limitation will likely apply to future retrospective or prospective studies of similar design. Nonetheless, our findings with respect to renal survival are in line with those of previous reports and did not change with our sensitivity analysis. Second, by virtue of having required a renal biopsy, our cohort is an inherently high-risk population, and findings may not be generalizable to all patients with DN. While this is an important consideration for our findings, large prospective studies evaluating the prognostic role of renal biopsy in otherwise healthy patients with DN are unlikely to be performed due to the morbidity associated with this procedure. Third, the lack of mortality data may have confounded our findings on renal survival, as risk of ESKD tends to be associated with risk of death, and censoring due to death may thus have been informative. However, this would not have affected our determination of predictors of eGFR slope. Finally, our assessment of renal histology was intentionally limited to histologic parameters described by the 2010 RPS classification. As a result, we cannot rule out that eGFR slope may be predicted by non-RPS histopathologic findings, such as ‘atubular glomeruli’ [26, 27], abnormalities in the glomerulotubular junctions with focal adhesions (‘tip lesions’) [26, 27], segmental sclerosis [20], extracapillary hypercellularity [20], hyaline caps [11], or microaneurysms [11].

Several studies have found that although eGFR loss varies significantly between patients, absolute eGFR loss in any given patient with diabetic nephropathy proceeds at a linear rate until the kidney ultimately fails [5–7]. Historically, in trying to identify rapid progressors, clinicians have focused on classic, easy to obtain risk factors for DN progression such as urinary ACR, blood pressure and glycemic control. Moreover, emphasis has traditionally been placed on single time point assessments of eGFR and, when available, histology. However, our data suggest that the best predictor of disease progression may in fact be serial eGFR measurements to establish baseline renal function and to enable calculation of an individual’s rate of eGFR loss. Indeed, eGFR slope was the most powerful predictor of ESKD in our cohort, with clinical risk factors such as urinary ACR and HbA_1c_ and histologic parameters such as IF/TA score providing no additive predictive value. Taken together, our data suggest that clinicians might benefit from placing more weight on obtaining serial eGFR measurements rather than traditional clinical risk factors or the RPS histological classification of DN.

## Conclusions

We found that histology, and the IF/TA score of the 2010 RPS classification system in particular, predicted ESKD in DN, but did not outperform serum creatinine-based eGFR measurements. Importantly, although multiple histologic features of DN have previously been found to predict the rate of renal function decline, this was not the case for any of the parameters included in the RPS histology score, which instead reflected only the burden of established disease. Other features absent from the 2010 RPS classification may still be predictive of eGFR loss, and thus merit further study. Of the parameters examined in this study, we observed that the best feature available to clinicians for prognostication of diabetic nephropathy is the slope of eGFR decline, as calculated from serial eGFR measurements. This parameter may in fact be more predictive than traditional risk factors such as urinary ACR and glycemic control, but requires at least some degree of patient monitoring. Larger studies are needed to validate the findings of this pilot study, and to identify novel and accurate predictors of rate of eGFR loss that more easily identify patients at risk for rapid progression to ESKD.

## Supplementary information

**Additional file 1: **Contains the following supplementary tables: **Supplementary Table 1.** Description of histology categories used in survival and regression analyses. **Supplementary Table 2.** Indication for biopsy. **Supplementary Table 3:** Comparison of multivariable Cox regression models. **Supplementary Table 4.** Sensitivity analysis for comparison of Kaplan-Meier Curves for ESKD-free survival, including decline in eGFR to below 15 ml/min in outcome. **Supplementary Table 5.** Sensitivity analysis for univariate Cox regression, including decline in eGFR to below 15 ml/min in outcome. **Supplementary Table 6.** Sensitivity analysis for multiple Cox regression, including decline in eGFR to below 15 ml/min in outcome.

## Data Availability

Summary data generated or analysed during this study are included in this published article [and its Supplementary Information files].

## Abbreviations

ACR: Albumin-to-creatinine ratio
DBP: Diastolic blood pressure
DN: Diabetic nephropathy
eGFR: Estimated glomerular filtration rate
ESKD: End-stage kidney disease
IF/TA: Interstitial fibrosis and tubular atrophy
RAS: Renin-angiotensin system
RPS: Renal Pathological Society
SBP: Systolic blood pressure
