# Supplementary material for: Renal histology in diabetic nephropathy predicts progression to end-stage kidney disease but not the rate of renal function decline
Source: BMC Nephrol. 2020 Jul 18;21:285. doi: 10.1186/s12882-020-01943-1 (PMC7368674; doi:10.1186/s12882-020-01943-1)
Supplement: Supplementary file 1 — Additional file 1: Contains the following supplementary tables: Supplementary Table 1. Description of histology categories used in survival and regression analyses. Supplementary Table 2. Indication for biopsy. Supplementary Table 3: Comparison of multivariable Cox regression models. Supplementary Table 4. Sensitivity analysis for comparison of Kaplan-Meier Curves for ESKD-free survival, including decline in eGFR to below 15 ml/min in outcome. Supplementary Table 5. Sensitivity analysis for univariate Cox regression, including decline in eGFR to below 15 ml/min in outcome. Supplementary Table 6. Sensitivity analysis for multiple Cox regression, including decline in eGFR to below 15 ml/min in outcome. [file 12882_2020_1943_MOESM1_ESM.docx]

**Supplementary tables**

**Supplementary Table 1: Description of histology categories used in survival and regression analyses**

| **Histology parameter** | **Study Class** | **Constituent RPS score(s) or class(es)** | **No. of patients without event** | **No. patients with event** |
| --- | --- | --- | --- | --- |
| Glomerular class | Low | I + II + III | 27 | 15 |
|  | High | IV | 3 | 5 |
| Interstitial fibrosis and Tubular atrophy score | Low | 1 | 13 | 3 |
|  | High | 2 + 3 | 17 | 17 |
| Arteriosclerosis score | Low | 0 + 1 | 15 | 8 |
|  | High | 2 | 15 | 12 |
| Arteriolar hyalinosis score | Low | 0 + 1 | 8 | 10 |
|  | High | 2 | 22 | 10 |

Legend: RPS, Renal Pathology Society·

**Supplementary Table 2: Indication for biopsy**

| **Indication** | **Number of patients (%)** |
| --- | --- |
| Proteinuria | 20 (40%) |
| Elevated or subacute rise in creatinine | 9 (18%) |
| Proteinuria and elevated creatinine | 5 (10%) |
| Other  -Hematuria, active urinary sediment, or glomerulonephritis  -Acute kidney injury  -Suspected multiple myeloma | 3 (6%)  2 (4%)  2 (4%) |
| Not available | 9 (18%) |

**Supplementary Table 3: Comparison of multivariable Cox regression models**

|  | **Covariate** | **Hazard ratio** | **Confidence interval** | **P value for hazard ratio** | **R^2^** | **P value for model** |
| --- | --- | --- | --- | --- | --- | --- |
| Model 1 (final) | Slope  -Per 5 ml/min/yr decrease  eGFR at biopsy  -Per 30 ml/min decrease | 2.15  6.48 | 1.59 – 2.92  2.29 – 18.3 | **<0.0001**  **0.0004** | 0.49 | **<0.0001** |
| Model 2 | Slope  -Per 5 ml/min/yr decrease  IF/TA Score  Class Low (ref)  -Class High | 2.05  13.66 | 1.52 – 2.79  2.08 – 89.9 | **<0.0001**  **0.007** | 0.43 | **<0.0001** |
| Model 3 | Slope  -Per 5 ml/min/yr decrease  eGFR at biopsy  -Per 30 ml/min decrease  IF/TA Score  Class Low (ref)  -Class High | 2.56  5.07  9.02 | 1.70 – 3.85  1.59 – 16.2  0.80 – 102 | **<0.0001**  **0.006**  0.08 | 0.53 | **<0.0001** |
| Model 4 (excluding slope) | ACR  -Per 100 mg/mmol increase  eGFR at biopsy  -Per 5 ml/min increase | 1.22  2.23 | 1.08 – 1.37  1.01 – 4.92 | **0.001**  **0.005** | 0.26 | **0.002** |

Legend: eGFR, estimated glomerular filtration rate; IF/TA, interstitial fibrosis and tubular atrophy; ACR, urinary albumin-to-creatinine ratio.

**Supplementary Table 4: Sensitivity analysis for comparison of Kaplan Meier Curves for ESKD-free survival, including decline in eGFR to below 15 ml/min in outcome**

| **Comparison** | **P value (log rank test for trend)** |
| --- | --- |
| Age (≤56 vs. >56 years) | 0.9 |
| Sex (male vs. female) | 0.2 |
| Slope (≤5.8 vs >5.8 ml/min/year) | **0.0001** |
| eGFR at biopsy ≤43.1 vs >43.1 ml/min) | **0.03** |
| ACR (≤216 vs >216 mg/mmol) | **0.003** |
| Hemoglobin A_1C_ (≤7.4 > 7.4%) | 0.7 |
| SBP (≤156 vs >156 mm Hg) | 0.4 |
| Glom (Low vs High) | 0.8 |
| IFTA (Low vs High) | **0.02** |
| Arteriosclerosis (Low vs High) | 0.3 |
| Arteriolar Hyalinosis (Low vs High) | 0.9 |

**Supplementary Table 5: Sensitivity analysis for univariate Cox regression, including decline in eGFR to below 15 ml/min in outcome**

| **Covariate** | **HR** | **95% Confidence interval** | **P value** | **R^2^** |
| --- | --- | --- | --- | --- |
| Age (per year) | 1.00 | 0.96 – 1.04 | 1.0 | 0.0 |
| Male Sex | 1.89 | 0.74 – 4.82 | 0.2 | 0.04 |
| Slope  -Per 5 ml/min/yr decrease | 1.37 | 1.15 – 1.63 | **0.0005** | 0.18 |
| eGFR at biopsy  -Per 30 ml/min decrease | 2.55 | 1.28 – 5.09 | **0.008** | 0.17 |
| ACR  -Per 100 mg/mmol increase | 1.21 | 1.09 – 1.36 | **0.0006** | 0.22 |
| Hemoglobin A_1C_  -Per 1% increase | 1.10 | 0.95 – 1.28 | 0.2 | 0.03 |
| SBP  -Per 10 mmHg increase | 1.01 | 0.97 – 1.03 | 0.5 | 0.001 |
| Glom (reference: Low)  -High | 1.12 | 0.33 – 3.82 | 0.86 | 0.001 |
| IFTA (reference: Low)  -High | 3.33 | 1.12 – 9.91 | **0.03** | 0.12 |
| Arteriosclerosis  (reference: Low)  -High | 1.61 | 0.69 – 3.72 | 0.3 | 0.03 |
| Arteriolar Hyalinosis  (reference: Low)  -High | 1.06 | 0.46 – 2.47 | 0.9 | 0.0 |

Legend: HR, hazard ratio; eGFR, estimated glomerular filtration rate; ACR, urinary albumin to creatinine ratio; SBP, systolic blood pressure; IFTA, interstitial fibrosis and tubular atrophy

**Supplementary Table 6: Sensitivity analysis for multiple Cox regression, including decline in eGFR to below 15 ml/min in outcome**

|  | **Covariate** | **Hazard ratio** | **Confidence interval** | **P value for hazard ratio** | **R^2^** | **P value for model** |
| --- | --- | --- | --- | --- | --- | --- |
| Model 1 (final) | Slope  -Per 5 ml/min/yr decrease  eGFR at biopsy  -Per 30 ml/min decrease | 1.82  8.81 | 1.41 – 2.35  2.60 – 29.8 | **<0.0001**  **0.0005** | 0.47 | **<0.0001** |
